# Long-term anti-predator learning and memory differ across populations and sexes in an intertidal snail

**DOI:** 10.1098/rspb.2024.0944

**Published:** 2024-10-09

**Authors:** Isabelle P. Neylan, Emily K. Longman, Eric Sanford, John J. Stachowicz, Andrew Sih

**Affiliations:** ^1^ Department of Evolution & Ecology, University of California, Davis, Davis, CA 95616, USA; ^2^ Center for Population Biology, University of California, Davis, Davis, CA 95616, USA; ^3^ Bodega Marine Laboratory, University of California, Davis, Bodega Bay, CA 94923, USA; ^4^ Department of Biological Sciences, Louisiana State University, Baton Rouge, LA 70803, USA; ^5^ Department of Environmental Science & Policy, University of California, Davis, Davis, CA 95616, USA

**Keywords:** *Nucella canaliculata*, predator–prey interactions, long-term memory, learning, anti-predator behaviour

## Abstract

Anti-predator behaviours in response to predator cues can be innate, or they can be learned through prior experience and remembered over time. The duration and strength of continued anti-predator behaviour after predator cues are no longer present, and the potential for an enhanced response when re-exposed to predator cues later is less known but could account for the observed variation in anti-predator responses. We measured the carryover effects of past predation exposure and the potential for anti-predator learning and memory in the marine snail *Nucella canaliculata* from six populations distributed over 1000 km of coastline. We exposed lab-reared snails to cues associated with a common crab predator or seawater control in two serial experiments separated by over seven months. Responses were population- and sex-dependent, with some populations retaining anti-predator behaviours while others showed a capacity for learning and memory. Male snails showed a strong carryover of risk aversion, while females were able to return to normal feeding rates and grow more quickly. These behavioural differences culminated in strong impacts on feeding and growth rates, demonstrating that this variation has implications for the strength of trait-mediated indirect interactions, which can impact entire ecosystems.

## 1. Introduction

Prey commonly exhibit anti-predator behavioural changes when exposed to cues associated with predators or predation [1–3]. These anti-predator responses often involve reduced activity leading to non-consumptive effects (NCE) that have costs in terms of reduced foraging time or mating opportunities. Furthermore, these behavioural modifications by prey alter their own effectiveness as predators, leading to trait-mediated indirect interactions (TMIIs) that can alter entire ecosystems [4–6]. Beyond innate responses to immediate threats, animals can use past experience to learn to associate cues with predation risk and thus pre-emptively deploy adaptive behavioural responses [7–9]. While there is ample evidence for priming of plastic anti-predator behaviour in nature, the capacity for learning and memory about cues associated with predation risk and the potential ecological implications are less well studied, particularly in non-vertebrate animals [10–12].

The adaptive advantage of learning and memory will depend on the environmental context and predation regime of a given population [13,14]. Learning should be selected for populations with variable and diverse predator regimes, whereas innate recognition of predators should dominate in areas with consistently high predator pressure or with low predator diversity [15]. Additionally, the state of the individual (e.g. size, sex, age and nutritional status) and their energetic demands should also influence what level of risk they are willing to tolerate and how sensitive they may be to risk [16,17]. Thus, the plasticity of risk aversion and the potential for anti-predator learning and memory across individuals should vary, either among populations from areas that vary in the abundance or identity of predators or food availability or among individuals with different metabolic demands based on age, reproductive status or sex.

Many animals exhibit strong, often innate anti-predator responses to alarm cues that emanate from distressed or harmed/deceased conspecifics [8,10,18]. Naive prey sometimes does not recognize predator cues (e.g. kairomones) as dangerous unless paired with an alarm cue, and in some species (such as many fish and amphibians), this kind of learning is required to associate kairomones with risk [19–21]. To assess whether animals have learned to associate kairomones with predation risk, a common approach involves simultaneously exposing an animal to a cue (e.g. a predator kairomone) paired with a clear indicator of predation risk (e.g. alarm cue). The investigator then quantifies how that earlier experience affects prey responses when later exposed to that cue alone (i.e. without the alarm cue; e.g. [22]). Learning is indicated if the animal exhibits an enhanced, appropriate response to the cue even in the absence of direct danger [23,24].

**Figure 1.**
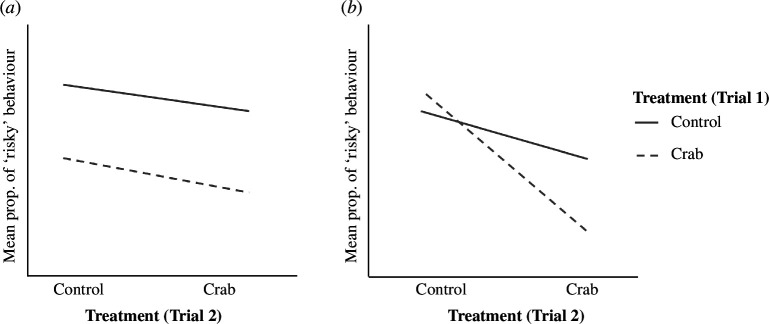
Graphs illustrating hypothetical behavioural responses (the mean proportion of *Nucella canaliculata* snails displaying ‘risky’ behaviour) to treatment combinations across trial 1 (indicated by line type) and trial 2 (indicated along the *x*-axis). (*a*) Potential carryover effect of predator exposure on the baseline levels of activity when no predator cues are present. This carryover effect is indicated because the dashed line is significantly lower than the solid line in trial 2 control treatments. (*b*) Possible predation risk learning and memory response, in other words, whether the response to crab cues in trial 2 depended on exposure to risk cues in trial 1. Learning is indicated because the slope of the dashed line (trial 1 crab treatment) is negative and steeper than the solid line (control trial 1 treatment) with the highest reduction in risk-taking behaviour when snails were exposed to crab cues twice (the right terminus of the dashed line).

Memory in this context involves the retention of alterations in behaviour even after the predator or alarm cue is no longer present [24]. We focused on the following two memory capabilities that are important in the context of predator–prey interactions: the carryover of risk aversion from past predator exposure (hereafter carryover or carryover effects) and the ability to learn and remember an adaptive response to predation (hereafter anti-predator learning and memory; figure 1). A carryover effect describes how long a fear response lasts after the threat or predator cue has passed as evidenced by a reduction in risk-taking behaviour even in safe, predator-free conditions (figure 1*a*). Individuals displaying a higher carryover capacity may benefit if earlier exposure to predators is a good predictor of later predation pressure by the same or similar predators [25]. These stronger carryover responses may be responsible for a stronger NCE and associated TMIIs [5,12,26]. Individuals that have less of this carryover effect can rebound faster to normal behaviours, such as foraging and mating, after the predator is gone and may even compensate for time and energy lost by more actively feeding or taking more risks when the environment appears safe [17,27]. Anti-predator learning and memory involves whether an individual continues to respond to a cue after they learn from an earlier exposure to associate that cue with risk indicated by reduced risk-taking when exposed to subsequent predator cues without a reduction in safe conditions (figure 1*b*). Individuals who retain the tendency to exhibit anti-predator behaviour in response to a given cue (e.g. a predator chemical cue) even in the absence of direct evidence of risk (e.g. the alarm cue) may be able to respond more effectively when predators return [15].

Snails, both freshwater and marine, have a well-documented suite of anti-predator traits including morphological and behavioural changes, and their behaviour is easy to observe and quantify in the laboratory, making them well suited to studies of learning and memory. These traits are often plastic in response to variation in predation risk [16,28–31], and previous experiences with predators can alter subsequent behaviour [10,12,29,32]. Anti-predator responses can be locally adaptive, and there is some evidence that the capacity to form memory over short time spans (hours or days) may also vary across populations of snails [13,33,34], but it is unknown how long these memories may last.

The channelled dogwhelk, *Nucella canaliculata*, is a species of marine snail that inhabits the rocky intertidal ecosystem and has documented anti-predator traits including changes in shell thickness and morphology as well as behavioural responses including fleeing, hiding and ceasing active foraging and feeding [35]. The *Nucella* genus of snails are mesopredators, feeding on sessile prey such as mussels and barnacles but also serving as prey themselves. Rock crabs (particularly *Cancer productus* and *Romaleon antennarium*) are common predators of *Nucella* spp. along the West coast of North America and induce anti-predator morphological and behavioural changes [35,36]. *Nucella* snails produce egg capsules with direct development and crawl-away juveniles, and the resulting low gene flow among populations increases the potential for adaptive divergence among populations [37,38]. In one study with *Nucella ostrina*, exposure to crab cues affected behavioural responses up to two weeks after crabs were removed [12]. In another study using *Nucella lamellosa*, prior crab and crushed-conspecific cue exposure reduced the threshold for anti-predator morphological response when exposed to crab cue alone two weeks later [39].

These findings suggest that *Nucella* snails may have the capacity for anti-predator learning, carryover from past predator exposure and predation risk memory; however, the time scale of documented effects has been relatively short. Thus, while strong trait-mediated indirect effects may rival or exceed density-mediated effects [40–42], the importance of TMIIs should decrease if behaviours rapidly return to normal once immediate predation subsides [26,43]. Indeed, most studies on snail memory only examine the retention of behaviour over hours or days [24,44]. For snails that may live 2–5 years, a longer duration of anti-predator carryover or memory could extend the impacts of these TMIIs far beyond what is currently appreciated, whereas a short duration would imply that the temporal scope of TMIIs is more limited. Furthermore, geographic variation in risk and other aspects of environmental conditions could result in variation in the capacity for learning and memory that alters the duration of TMIIs and their relative importance across a landscape [5,16,45].

To address this possibility, we ran a laboratory experiment using lab-reared *N. canaliculata* snails as the focal prey species. We assessed any potential for learning and memory over a seven-month period both in terms of legacy effects on baseline activity (carryover effects) and the strength of response when exposed to predator cues again (anti-predator learning and memory) and assessed whether responses differed among populations and between sexes. We used these results to assess whether environmental context and energetic and life history constraints impose differential selection on learning and memory formation.

## 2. Methods

### (a) Experimental overview

We ran a fully crossed, factorial experiment that exposed *N. canaliculata* snails to either control or predator cue treatments (rock crabs with crushed-conspecific snail cues) in an initial experiment (trial 1) followed seven months later by another experimental exposure (trial 2) to either a seawater control or predator cues (rock crabs, no crushed conspecific). During both of these exposures, we tracked each individual snail’s behaviour in containers that provided areas of refuge and risk. This design allowed us to assess (i) how prior exposure to predator cues influenced the behaviour of snails months later in the absence of additional predator cues (carryover effects), (ii) how prior experience influenced the behaviour of snails in a subsequent exposure to predator cues (anti-predator learning/memory), and (iii) how these capabilities varied depending on source population or sex.

### (b) Organism collection and maintenance

**Figure 2.**
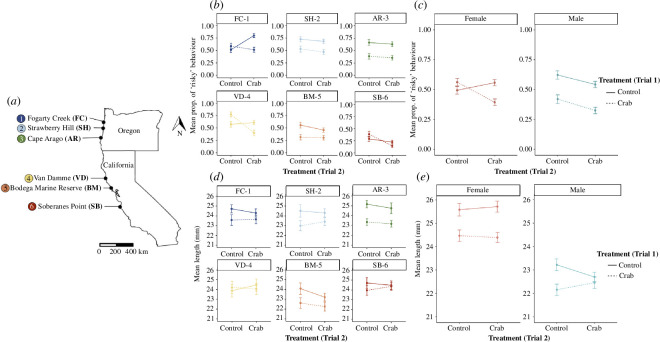
(*a*) Map of the six populations along the coast of California and Oregon, USA, where *N. canaliculata* were collected for laboratory experiments. The mean proportion of snails displaying ‘risky’ behaviour (out in the open or feeding) averaged over 5 days in trial 2 across (*b*) treatments and populations, and (*c*) treatments and sex (female versus male). The mean length (mm) of snails immediately prior to the start of trial 2 across (*d*) treatments and populations, and (*e*) treatments and sex. For all graphs, trial 2 treatments are along the *x*-axis and the line type refers to trial 1 treatments (solid lines indicate control, and dashed lines indicate crab treatment). Error bars represent standard error.

In the summer of 2019, we collected egg capsules from three sites along the Oregon coast: Fogarty Creek (FC-1; 44° 50′ 17.72″ N, −124° 3′ 34.96″ W), Strawberry Hill (SH-2; 44° 15′ 19.40″ N, −124° 6′ 42.52″ W) and Cape Arago State Park (AR-3; 43° 18′ 23.18″ N, −124° 23′ 55.14″ W), and three sites along the California coast: Van Damme State Park (VD-4; 39° 16′ 59.72″ N, 123° 47′ 49.69’ W), Bodega Marine Reserve (BM-5; 38° 19′ 5.95″ N, −123° 4′ 20.28″ W) and Soberanes Point (SB-6; 36° 27′ 14.90″ N, −121° 55′ 43.90″ W; figure 2*a*). We sampled from across six populations covering over 1000 km of coastline to try to capture variation in an ecological context, particularly predation regime and access to refuge, and to see how widespread memory and learning patterns are in this snail. In general, the Oregon coast (especially FC-1 and SH-2) has denser, less open mussel matrices with fewer places to seek refuge as well as higher predation rates than the Northern California coast (VD-4, BM-5 and SB-6; I.P.N., E.K.L. & E.S. 2019, personal observation) [46]. While we tried to standardize environmental factors such as wave exposure and elevation across sites, other factors such as temperature and pH differ across sites potentially impacting patterns. However, we raised all snails in common garden conditions (see details below) to lessen some of this variation among the populations.

We collected 11–15 sets of egg capsule clusters at each site with each cluster separated by at least 1 m to minimize relatedness between ‘families’ of snails across an area of about 20–50 m^2^ at each site. We brought egg capsules back to the Bodega Marine Laboratory in Bodega Bay, CA, and placed them into fine mesh containers in flow-through seawater until juvenile snails emerged and began feeding. Once large enough, we moved the snails into 1 l plastic containers grouped by family (snails originating from the same cluster of egg capsules) and population with no more than 64 snails per container and maintained them in a flow-through seawater system. We fed the snails *Mytilus trossulus* mussels ad libitum until they reached adulthood and were ready to be used in experiments. Rearing the snails to adulthood in the lab allowed us to control the snail’s exposure and experience with predators.

Once the snails reached adulthood (about 1.5 years) in the winter of 2021, we determined the sex of each snail by visual inspection and gave each snail a unique identifying tag (Betterbee bee tags) affixed with super glue (Loctite super glue gel control) to their shell. The tags allowed us to track individual snails throughout experiments and between trials. Prior to both experimental trials and after the initial 12 weeks of trial 1, we measured shell length (mm) using callipers, live/wet mass (g) using a standard balance and calculated weight-to-length ratios as these are good indicators of size and have been used in previous work with *Nucella* [12,47,48]. Length and weight were highly correlated (linear regression; *F*(1,441) = 1020, adjusted *R* ^2^ = 0.6975, *p* < 0.001), and patterns were qualitatively the same in subsequent analyses using either length, weight or the weight-to-length ratio so here we report only analyses using length as our metric for overall size.

### (c) Experimental design

We conducted experiments in a flow-through seawater system (with fresh seawater continuously supplied) consisting of three vertical levels. On each level, we elevated four plastic containers that each received independent seawater inflow. These containers then gravity-fed water into manifolds that supplied seawater to each individual experimental arena (electronic supplementary material, figure S1). These elevated containers were either left empty (with just ambient seawater) as a control or housed rock crabs (see more details for both trials below).

The experimental arenas consisted of 1 l cylindrical plastic containers with holes drilled halfway up the sides of the container and covered with fine mesh allowing water to drain without the snails escaping. To deliver water to each experimental container, we passed tubing from the elevated crab or control containers through a hole in the secured lid of each experimental container. This design ensured that each container was filled only halfway with seawater giving snails the ability to escape the water as one possible refuge [12,36]. We also included an apparatus in each container (electronic supplementary material, figure S1) that forced snails to choose between either accessing mussels in a vulnerable location (i.e. in a submerged, open dish) or hiding in a safe refuge under the dish with no food (i.e. creating a trade-off between access to food and safety) [49,50].

For all trials, we measured behaviour by documenting the location of each individual snail in the experimental arena during weekly intervals for trial 1 and daily for trial 2. We then condensed these measures into binary categories, ‘safety’, which included snails positioned out of the water or hidden, or ‘risk’, which included snails in the water but out in the open and/or snails actively feeding. Every day that we conducted behavioural observations, we checked every container and documented every snail within the same several hour period. We randomized the starting location of these checks within the sea table to minimize any potential biases due to order or timing. We also replaced food (*M. trossulus*) once a week (trial 1) to ensure there was consistent motivation for snails to leave the refuge and counted the number of mussels eaten within each container weekly (trial 1).

### (d) Trial 1

For trial 1, we placed a pair of snails, one male and one female, from the same population but a different family (with 10–13 families per population equally distributed across treatments), into each experimental arena. In this trial, we used rock crabs (either *C. productus* or *Romaleon antenarium*) fed crushed *N. canaliculata* within the elevated container as the predator and alarm cue treatment in half of the elevated containers, while keeping the other half empty as the controls. To ensure there were no differences in response to the two crab species, we conducted two additional behavioural observations during the first week of the experiment for a total of three observations across 6 days noting the species of crab in each elevated container. We found no difference in snail reaction to the two species of rock crab (GLMM; *p* = 0.324, Df(*N*) = 1(867)). In addition, crabs were rotated weekly to reduce any individual crab or species-level differences. We collected crabs from Bodega Harbor in Bodega Bay, CA (38° 19′ 5.95″ N, −123° 4′ 20.28″ W) and all were adults over the legal catch size limit (greater than 10.16 cm, carapace width). Crabs were kept in the system for 3 days at a time and then removed for 4 days before being replaced again to encourage female snails to lay egg capsules as part of a separate experiment. All behavioural observations in this analysis were conducted on the third day of the crabs being present in the system. These observations were recorded once a week for 12 weeks from March to May of 2021.

After this 12-week trial, the snails remained in cue (crab or control) under the same conditions for an additional eight weeks (a total of 20 weeks in cue). We then transferred all snails into storage containers, which consisted of the same 1 l plastic containers but mesh outflows on the lids of the containers rather than the sides allowing them to fill completely, and we removed the behavioural apparatuses. Each container housed about 48 snails from the same population that had experienced the same treatment. All snails received ambient, flow-through seawater without predator or alarm cues for 29 weeks and continued to be fed *M. trossulus* ad libitum allowing for potential compensatory growth across treatments.

### (e) Trial 2

After 29 weeks of ambient, no predator or alarm cue conditions, we began trial 2 in February of 2022. We used the same sea table set-up, but in this trial, we used only red rock crabs (*C. productus*) as the predator cue without crushed conspecifics (crabs were fed sardines prior to the experiment and were not fed within the elevated containers). As before, half of the elevated containers had crabs while the other half were left empty as the controls, but in this trial, we had only five elevated containers, two controls and three with crabs due to space constraints. We placed five individually marked snails in each experimental arena. Each arena had snails from the same population and trial 1 treatment and had at least two males and two females. This trial ran for 7 days total. We introduced snails into the system on day 1 without any cues, added crabs on day 2 and took daily behavioural observations from day 3–7 (five observations total).

### (f) Statistical analyses

For all analyses, we only included snails that were present in both trial 1 and trial 2. Some mortality occurred over the 29 weeks between trials, with higher mortality on average in snails that had experienced crab cues than control cues and in males than females (electronic supplementary material, table S2). This left a total of 443 snails with 12 observations per individual in trial 1 and five observations per individual in trial 2. Sample sizes per population in trial 1 ranged from 31 to 42 and from 14 to 28 in trial 2 (see electronic supplementary material, table S1 for full replication details of each treatment combination). For sex, sample sizes ranged between 94 and 123 per treatment in trial 1 and between 41 and 73 in trial 2 (electronic supplementary material, table S1).

For trial 1, we analysed behavioural data using a generalized linear mixed effect model assuming a binomial distribution (logit link function). We considered treatment (crab or control), population, sex and the interactions between these factors including a three-way interaction as fixed factors. We found that the three-way interaction was not significant, and removing it improved our Akaike information criterion (AIC) score, so we removed these interactions from subsequent analyses. We included the individual snail, family identity and experimental container used during the trial as random factors. We used the same model formulation and analyses for our behavioural data in trial 2 but included trial 1 treatment (crab or control), trial 2 treatment (crab or control), population, sex and all interactions including a four-way interaction as fixed factors, but ultimately dropped the four-way interaction as it was not significant and improved the model fit (AIC score). Trial 2 container, individual snails and families were used as random factors. We conducted post hoc pair-wise comparisons of treatment effects within populations or within sexes from these full models. We also ran separate models for all populations and each sex to determine treatment differences within each group rather than across groups as this was our primary objective.

We analysed feeding rates during trial 1 by using the total number of mussels eaten in each experimental container as the response variable and running a generalized linear mixed effects model using a negative binomial distribution (log link function) given that our response was count data that included zeros and was over-dispersed. Treatment (crab or control), population and the interaction between these two factors were considered fixed factors, and family and experimental container were considered random factors. We could not include sex in these models as feeding rates were measured per experimental container that contained one male and one female with no way to determine which snail ate which mussels. We estimated snail growth during trial 1 by using change in length (mm) calculated by subtracting each snail’s starting length from their final length at week 12 as our response variable. We analysed snail growth using a generalized linear model assuming a Tweedie distribution as our data were continuous but zero-inflated and did not involve negative numbers (snails did not shrink) [51]. We considered treatment, sex, population and their interactions as fixed effects, but we again dropped the three-way interaction between treatment, sex and population as it was not significant and improved model fit. The model failed to converge when random effects were included, so we continued with this simplified model.

Finally, we wanted to assess whether the size of individual snails after trial 1 and the ambient conditions period affected behavioural patterns in trial 2. First, we assessed whether size differed among the snails prior to the start of trial 2 using a linear mixed effect model with a Gaussian distribution on length prior to trial 2 with trial 1 treatment, sex, population and their interactions as fixed effects (dropping the non-significant three-way interaction) and including family as a random factor. Additionally, we ran a series of simple linear regressions to test if size significantly predicted behaviour across our trials.

We ran all of our generalized linear mixed effect models using the glmmTMB package [52] and assessed the significance of fixed effects by analysis of deviance, type III Wald chi-square tests using the ANOVA function from the car package [53]. We explored pair-wise differences using a Bonferroni correction and the ‘contrasts’ function in the emmeans package [54]. For linear regressions, we used the lm package [55]. All analyses were performed in R version 4.4.0.

## 3. Results

### (a) Trial 1—behavioural response

All populations decreased risk-taking behaviour in the presence versus absence of crabs, but the degree of that reduction differed among populations as evidenced by a significant treatment by population interaction (*p* = 0.007; table 1a; electronic supplementary material, figure S2A). Populations all had similar levels of risk-taking behaviour in the crab treatment but differed in boldness in the control treatment, with FC-1 and VD-4 tending to be bolder while SB-6 tended to be the most risk-averse (pair-wise comparisons; electronic supplementary material, table S3). The snails that had the biggest relative reduction in risk-taking behaviour between control and predator treatments were SH-2 (42.9% reduction in risky behaviour) and AR-3 (47.6%), while VD-4 had the lowest reduction (30.4%). FC-1, BM-5 and SB−6 fell in between (34.2, 34.1 and 39.8%, respectively). Both males and females had a similarly strong response to the predator cues (no significant sex by treatment interaction, *p* = 0.441), but females displayed more risky behaviour across treatments (significant effect of sex *p* = 0.017; table 1a; electronic supplementary material, figure S2B).

**Table 1.** Model results examining (*a*) the effect of predator cue exposure, population and sex on the relative proportion of snails displaying ‘risky’ behaviour during trial 1, (*b*) the effect of predator cue exposure and population on feeding rate (total number of mussels eaten over the course of the trial) during trial 1, (*c*) the effect of predator cue exposure, population and sex on growth (change in length) during trial 1, and (*d*) the effect of predator cue exposure, population and sex on size (length) prior to the start of trial 2.

(*a*) behaviour trial 1				(*b*) feeding rate trial 1			
fixed effects	Df	chi-square	*p*	fixed effects	Df	chi-square	*p*
treatment	1	354.726	**<0.001**	treatment	1	456.896	**<0.001**
population	5	31.160	**<0.001**	population	5	47.016	**<0.001**
sex	1	5.696	**0.017**	treatment × population	5	12.819	**0.025**
treatment × population	5	16.055	**0.007**	**random effects**	variance	s.d.	
treatment × sex	1	1.623	0.203	container	<0.001	<0.001	
population × sex	5	4.8	0.441	family	<0.001	<0.001	
**random effects**	variance	s.d.					
individual	0.189	0.435					
container	0.273	0.523					
family	0.034	0.184					

(*c*) growth rate trial 1				(*d*) size prior to trial 2			
fixed effects	Df	chi-square	*p*	fixed effects	Df	chi-square	*p*
treatment	1	335.245	**<0.001**	treatment	1	42.860	**<0.001**
population	5	48.001	**<0.001**	population	5	24.725	**<0.001**
sex	1	41.697	**<0.001**	sex	1	275.565	**<0.001**
treatment × population	5	15.793	**0.007**	treatment × population	5	6.787	0.237
treatment × sex	1	0.546	0.460	treatment × sex	1	3.415	0.065
population × sex	5	7.626	0.178	population × sex	5	29.887	**<0.001**
				**random effects**	variance	s.d.	
				family	0.271	0.520	

The effect of *p*-values with significant results (*p* < 0.05) are shown in bold.

### (b) Trial 2—carryover effect

To assess the potential carryover effect of predator exposure on the baseline levels of activity when no predator cues are present, we compared the behaviour of snails with differing previous exposure treatments (from trial 1) in the control treatment of trial 2 (figure 1*a*). We found a significant three-way interaction between population, trial 1 treatment and trial 2 treatment (*p* = 0.049), indicating that populations differed in the amount of carryover from trial 1. Population-specific models found significant effects of crab treatment across most of our populations, but which trial or combination of trial treatments had significant effects varied (figure 2*b*; electronic supplementary material, tables S4 and S5). In particular, we found that in AR-3 snails, exposure to predation cues caused reduced activity in snails as compared to their naive counterparts as evidenced by trial 1 treatment effects (figure 2*b*; electronic supplementary material, table S4; *p* < 0.001, AR-3) and that snails significantly reduced their risk-taking behaviour when previously exposed to crab cues as compared to their naive counterparts in the trial 2 control treatment (figure 2*b*; electronic supplementary material, table S5). Snails from SH-2 showed similar patterns, but these results were marginally non-significant (figure 2*b*; electronic supplementary material, table S4; *p* = 0.051). In contrast, snails from BM-5, FC-1, VD-4 and SB-6 did not differ significantly in behaviour due to trial 1 treatment, and there was no significant difference between snails in trial 2 control treatments that had or had not experienced crab exposure in trial 1 and therefore do not appear to experience a carryover from past predator exposure that impacts their baseline behaviour (figure 2*b*; electronic supplementary material, tables S4 and S5).

We also found a trial 1 treatment × sex interaction on behaviour in trial 2 (table 2; *p* = 0.012) suggesting differences between the sexes in carryover capacity. Males tended to show stronger retention of risk aversion after crab exposure as supported by an effect of trial 1 treatment (*p* < 0.001; electronic supplementary material, table S4) and a significant reduction in risk-taking behaviour after exposure to crab and alarm cues in trial 1 even in safe conditions in trial 2 (figure 2*c*; electronic supplementary material, table S5). Overall, males were more risk-averse than females, and fear was retained even in control conditions seven months later. Females, on the other hand, did not show this pattern of carryover (figure 2*c*; electronic supplementary material, tables S4 and S5). There were no interactions involving sex and population (table 2; *p* = 0.658), indicating that male–female differences were similar across populations.

**Table 2.** Model results examining the effect of predator cue exposure in trial 1 and trial 2, population and sex on the relative proportion of snails displaying ‘risky’ behaviour during trial 2.

fixed effects	Df	chi-square	*p*
treatment-trial 1	1	13.030	**<0.001**
treatment-trial 2	1	1.569	0.210
population	5	67.178	**<0.001**
sex	1	0.665	0.415
treatment-T1 × treatment-T2	1	1.755	0.185
treatment-T1 × population	5	11.868	**0.037**
treatment-T2 × population	5	9.827	0.080
treatment-T1 × sex	1	6.248	**0.012**
treatment-T2 × sex	1	0.001	0.970
population × sex	5	3.270	0.658
treatment-T1 × treatment-T2 × population	5	11.083	**0.049**
treatment-T1 × treatment-T2 × sex	1	2.209	0.137
**random effects**	variance	s.d.	
individual	0.082	0.297	
container	1.33	1.154	
family	<0.0001	0.0002	

*P*-values with significant results (*p* < 0.05) shown in bold.

### (c) Trial 2—anti-predator learning and memory

To test for predation risk learning or memory, we asked whether the response to crab cues in trial 2 depended on exposure to risk cues in trial 1, in other words, a trial 1 × trial 2 interaction. More specifically, we also looked for whether there was a greater reduction in risk-taking behaviour between trial 2 control and crab when a snail had previous exposure to crab cues as compared to their naive counterparts (as illustrated by a steeper negative slope; figure 1*b*), as this would indicate not only a capacity for memory across trials but that the memory led to more optimal behavioural responses.

The interaction between trial 1 and trial 2, however, depended on population (table 2; *p* = 0.049), indicating that populations differed in memory patterns. In three of our six populations, there was little evidence for a consistent learning or memory effect with no significant interaction term between trial 1 and trial 2 treatments and no pattern of a stronger behavioural change across trial 2 treatments between previously exposed and naive snails (SH-2, AR-3 and BM-5; figure 2*b*; electronic supplementary material, tables S4 and S5). Trial 1 × trial 2 treatment did interact in two populations, FC-1 and VD-4 (electronic supplementary material, table S4; *p* = 0.033, FC-1; *p* = 0.032, VD-4). In VD-4 snails, we saw a strong reduction in risk-taking behaviour in the crab–crab treatment (figure 2*b*) without a corresponding reduction in crab–control treatments suggesting anti-predator memory (electronic supplementary material, table S5; *p* = 0.024). In contrast, in the FC-1 population, this interaction is probably driven by snails displaying the riskiest behaviour in the control–crab treatment, which would not be an optimal response (electronic supplementary material, table S3). Finally, it is worth noting that SB-6 snails appear to have a similar behavioural pattern to that of VD-4 that would suggest possible fear memory (figure 2*b*), but they lacked a significant trial 1 by trial 2 interaction term (electronic supplementary material, table S4; *p* = 0.251).

Female snails showed a potential fear memory response as supported by a significant trial 1 × trial 2 interaction (electronic supplementary material, table S4; *p* = 0.029). This finding is likely driven by a reduction in risky behaviour in the crab–crab treatment as compared to the control–crab treatment, which further supports adaptive fear memory (electronic supplementary material, table S5; *p* = 0.039). However, there was not a significant trial 1 × trial 2 × sex interaction in the full model indicating that males and females did not significantly differ in their responses (table 2; *p* = 0.137; figure 2*c*).

### (d) Feeding, size and growth

During trial 1, we found a significant interaction between treatment and population in feeding rate (table 1*b*; *p* = 0.025; electronic supplementary material, figure S3). While populations ate at similar, higher rates in control conditions, the interaction was driven by differences between populations in the crab treatment (electronic supplementary material, table S6). We also found a significant interaction between population and treatment (*p* = 0.007) on the growth rates of snails in trial 1 (as measured by the change in length over the 12 weeks of the experimental period; table 1 and electronic supplementary material, figure S4). This interaction was also entirely driven by differences between populations in the crab treatment with no significant population differences in control treatment (electronic supplementary material, table S7). Sex was also significant in our model (table 1; *p* < 0.001). Females grew over 1.5 times more than males across treatments (average female growth rate 1.79 ± 0.11 mm; average male growth rate 1.01 ± 0.074 mm).

These differences in feeding and growth rates during trial 1 appeared to carry forward and affect sizes of snails prior to the start of trial 2 even with the long (29 weeks) period in ambient conditions and ad libitum feeding. We found a significant effect of treatment (table 1d; *p* < 0.001) on snail size with snails in control conditions in trial 1 larger than snails that experienced crab treatment (figure 2*d*,*e*). We also found significant effects of population (*p* < 0.001), sex (*p* < 0.001) and population by sex interaction (*p* < 0.001; table 1 and figure 2*d*,*e*). Female snails tended to be larger than male snails on average, but the magnitude of this difference varied across populations (electronic supplementary material, table S8).

We did not find a significant correlation between size prior to a trial (1 or 2) and subsequent risk-taking behaviour across our series of linear regressions. First, we found no significant correlation between size prior to trial 1 with subsequent behaviour in trial 1 (electronic supplementary material, figure S5). Size after trial 1 did correlate with behaviour during trial 1 across all snails as well as within each treatment (control or crab), and there was a significant positive correlation with bolder snails growing more across all snails and within each treatment (electronic supplementary material, figure S6). We also examined whether size prior to trial 2 impacted subsequent behaviour in trial 2 within each trial 1 and trial 2 treatment combination (control–control, control–crab, crab–control and crab–crab). We did not find any significant correlations between size and behaviour in any of the treatment combinations (electronic supplementary material, figure S7). We went one step further and also ran regressions for each trial 1 treatment across all populations and both sexes and found all were non-significant (*p* > 0.05) except for VD-4 snails that experienced control conditions in trial 1 (*p* = 0.002; adjusted *R* ^2^ = 0.202) and a marginally significant correlation in female snails that experienced crab conditions in trial 1 (*p* = 0.046; adjusted *R* ^2^ = 0.027), and marginally non-significant in those that experienced crab cues (*p* = 0.057; adjusted *R* ^2^ = 0.022).

## 4. Discussion

We found evidence for both carryover from past predator exposure and anti-predator learning and memory over a seven-month period in an intertidal marine snail, but these patterns differed across populations and sexes. First, across all snails, we found strong evidence of an innate ‘fear’ response to chemical cues from predators when coupled with alarm cues from crushed conspecifics (trial 1), but this response did not hold true for crab cues alone (trial 2). We found that in one population (VD-6) snails were capable of learning to associate crab cues with predation risk and then reacting to the presence of predator cues alone in future exposures while in another population (AR-3), and in male snails, the prior experience carried over into subsequent behaviour even in the absence of kairomones. Importantly, these behavioural patterns corresponded to differences in feeding rate in trial 1, which affected individual growth and impacted the size of snails even seven months later and suggests that these changes in behaviour can have meaningful impacts on the consumption of lower trophic levels.

We observed a carryover effect in some groups (AR-3 and males) that may be indicative of the lasting effects of predator exposure on future behaviour. This retention effect has been documented in other snail experiments, although over much shorter timescales (days or weeks rather than months) [10,12,32], as well as in other aquatic systems that use chemosensory predator and alarm cues such as tadpoles [56] and fish [57,58]. This more enduring and conservative approach to risk may come at the cost of feeding and mating opportunities [59]. A longer-lasting predator legacy effect would strengthen TMIIs and may have cascading trophic effects with lower predation rates on basal resources such as mussels and barnacles impacting intertidal invertebrate cover [12,33,38,45,60]. Snails originating from AR-3 also had one of the strongest innate responses to crab and alarm cues in trial 1. One untested explanation is that this population experiences the highest and/or most consistent crab predation risk, which would select for the strongest innate responses, and therefore we would expect little indication of learning [22].

In terms of learning and memory, we found that snails across populations and sexes exhibited a significant trial 1 treatment effect on behaviour in trial 2, suggesting that snails learned from prior experience that crab cues indicate predation risk. However, importantly, this response depended on the population. Only one population, VD-4, showed true evidence for a learning and memory response with a significant trial 1 × trial 2 interaction as well as a significant difference in behaviour between crab–control and crab–crab treatments in trial 2 (dashed line; figures 2*b* and 1*b*) with a corresponding lack of significant difference across naive snails (solid line; figures 2*b* and 1*b*). FC-1 snails also showed a significant trial 1 × trial 2 treatment interaction; however, looking at their behaviour we see that this result is largely driven by crab-naive snails displaying the riskiest behaviour when in the presence of crab cues, a suboptimal response. SB-6 snails show a very similar pattern to VD-4, but without significant differences perhaps driven by their lower risk tolerance overall. Interestingly, along with learning and remembering to avoid risk in the presence of kairomones, VD-4 snails also show a rebound effect, meaning that they increased their risk-taking in safe, control conditions after prior exposure to a threat in trial 1 suggesting that this population may be the most attuned or sensitive to predator cues and able to modulate behaviour most effectively. A diverse predation regime or one that varies enough to make risk tracking a more valuable asset would be one possible factor favouring this response [45,61,62]. Previous studies have found population-level differences in responsiveness to risk cues in *Nucella* snails correlated with the relative predation risk at their site of origin [33,63,64]. Another possibility is that there may be population-level differences in how resources need to be allocated and thus differences in the assessment of risk [16,65]. Previous work with these same six populations of *N. canaliculata* has shown strong population-level differences in diet preference and ability to drill into thick mussel shells [36] (Longman & Sanford submitted). In particular, SB-6 and VD-4 snails have the capacity to drill large mussels (*Mytilus californianus*) with thick shells, which can take days of dedicated time as opposed to Oregon populations, which exploit smaller mussels and barnacles that can be harvested much more quickly. There could be a strong benefit to being able to detect danger and take full advantage of safe conditions as feeding is potentially riskier and more time-consuming for populations of snails exploiting these larger prey sources. Further work quantifying the predation risk and environmental conditions in the field at these sites would be valuable in fully exploring conditions that promote these different learning strategies. Additionally, experiments testing behavioural responses to predator cues only and/or conspecific alarm cues only along with the predator and alarm cue combination during the first trial would also help untangle true legacy and memory effects and provide further evidence of learning.

Males and females differed in carryover and memory responses (figure 2*c*). Males exposed to crabs in trial 1 reduced their risky behaviour in trial 2 in both control and crab treatments, so this carryover effect seems to overwhelm any possible learning or memory effect. Females were bolder in trial 1 and lacked a carryover effect in trial 2, suggesting a difference in risk tolerance across male and female snails and across. For female snails, one possible explanation for limiting fear responses may be related to the risk allocation hypothesis and the need to maximize feeding in order to reproduce [16,59,65]. Given the importance of energy intake for fecundity in *Nucella* [66], it might be more costly for females than males to over-avoid risk cues and thus be adaptive to resume feeding relatively quickly after a predator has left the area or risk is no longer perceived [17]. Over the course of the 12 weeks in trial 1, we found that females grew significantly more than males in both crab and control treatments. Importantly, trial 1 occurred during the breeding season for these snails, and females were actively laying egg capsules throughout this trial (although not enough females laid viable eggs across treatments for statistical analyses).

Beyond learned memory effects, there is the possibility that differences in size, individual condition, metabolism and other factors may also carry forward and impact behavioural patterns. In trial 1, we found evidence that higher proportions of risk-taking behaviour were generally correlated with higher feeding rates, which led to higher growth rates, and ultimately post-trial 1 size was significantly correlated with increased risk-taking behaviour during trial 1. We see similar patterns across behaviour, feeding rate and growth rate throughout trial 1. This highlights the potential for behavioural changes in response to predation to have ecosystem-level consequences through TMIIs by directly affecting feeding rates. However, it also poses a challenge to fully untangle whether the effects we see from the trial 1 treatment on trial 2 behaviour are due to memory alone or a size or condition carryover (snails in trial 1 control treatment grew more than those in crab treatment). While differences in size and previous feeding rates may be playing a role in subsequent behaviour, we believe there is still evidence for behavioural carryover, learning and memory as size alone did not predict boldness within treatments in trial 2 (electronic supplementary material, figure S7) and trial 1 treatment did not significantly interact with either population or sex in our size prior to trial 2 analysis (table 1). Furthermore, the seven months of ambient conditions between trial 1 and 2 with readily available food allowed considerable time for snails to compensate for any nutritional deficits they may have experienced when exposed to crab cues in trial 1. Regardless of the mechanism, it is clear that prior experience had an important and long-lasting impact on subsequent behaviour in these snails and that these patterns were consistently different across populations and sexes over an extended period of time. Measuring multiple aspects of an individual (behaviour, size and individual status) and considering them all as part of an integrated phenotype shaped by their experience or lack of experience with predators is an important consideration to fully explore the impacts of predator–prey interactions.

This study, to our knowledge, is the longest test of memory (over seven months of retention) in snails to date. However, there is a relatively robust literature investigating memory formation in gastropods. These studies have demonstrated that snails can develop associative networks between a given stimulus (crab cue in our case) and a risk cue [23,24]. Historically, this capacity was thought to only exist in vertebrates [39]. Studies in the pond snail *Lymnaea stagnalis* have demonstrated a consistent ability for snails to achieve configural learning, the ability to respond to two cues when experienced in combination but not individually, although over shorter time frames (hours or days) [24,44,67,68]. Other memory experiments in *Lymnaea,* such as appetitive or food-reward conditioning, may last over two weeks [69,70] and sustained operant training for breathing behaviour in hypoxic conditions can elicit memory even a month later [71]. Though the underlying mechanisms of this memory in our experiment are unclear, long-term memory formation in other gastropods (*Lymnaea* and the sea hare *Aplysia californica*) occurs through a combination of changes in gene expression, creation of RNA, protein synthesis and the formation of new synaptic connections [69,72,73].

Anti-predator behaviour allows animals to dynamically respond to predation risk. While many studies have shown that evolutionary and ecological forces can shape the innate strength of the behavioural response, we have also demonstrated that the longevity and sensitivity of the response may be affected. As a meso-predator, *N. canaliculata* can influence food web dynamics and community structure as both a prey and a predator, influencing the abundance of foundation species such as mussels and barnacles [38,45,60]. For populations such as AR-3 with lasting fear legacy effects on behaviour and ultimately feeding rates, cascading effects of predator presence could be greater than anticipated, as the mere presence of a few predators may be sufficient to induce lasting TMIIs. Conversely, cascade strength may be weaker in populations such as VD-4 that can more dynamically respond to predator presence or absence, limiting the duration of TMIIs. Our findings contribute to the growing body of evidence in NCE and TMII literature, emphasizing the importance of understanding the ecological context and the state of individual prey and predators to fully predict ecosystem-wide impacts of predator–prey interactions [4,5,16]. The ability to learn when and for how long to display risk-averse, anti-predator behaviour may be another important facet within a suite of anti-predator traits. The amount and variability of predation risk and the condition of the individual and their energetic needs may all play a part in dictating whether fear memory and retention are adaptive or maladaptive and ultimately impact the effect of predators and the strength of trophic cascades.

## Data Availability

The data have been deposited and are available through Dryad [74]. Supplementary material is available online [75].
